# Pharmacological Activities of* Alisma orientale* against Nonalcoholic Fatty Liver Disease and Metabolic Syndrome: Literature Review

**DOI:** 10.1155/2019/2943162

**Published:** 2019-06-03

**Authors:** Eunsol Choi, Eungyeong Jang, Jang-Hoon Lee

**Affiliations:** ^1^Department of Clinical Korean Medicine Graduate School, Kyung Hee University, 26 Kyungheedae-ro, Dongdaemun-gu, Seoul 02447, Republic of Korea; ^2^Department of Internal Medicine, College of Korean Medicine, Kyung Hee University, 26 Kyungheedae-ro, Dongdaemun-gu, Seoul 02447, Republic of Korea; ^3^Department of Internal Medicine, Kyung Hee University Korean Medicine Hospital, 23 Kyungheedae-ro, Dongdaemun-gu, Seoul 02447, Republic of Korea

## Abstract

Nonalcoholic fatty liver disease (NAFLD) is a rapidly emerging hepatic manifestation of metabolic syndrome. However, its unrevealed mechanism and complicated comorbidities have led to no specific medication, except for weight loss and lifestyle modification.* Alisma orientale* (Sam.) Juzep (*A. orientale*, Alismataceae) has been increasingly reported on therapeutic effects of* A. orientale* against NAFLD and metabolic syndrome such as insulin resistance, hyperlipidemia, and obesity. Therefore, this study aimed to review the preclinical efficacy of* A. orientale* and its chemical constituents including Alisol A 24-acetate, Alisol B 23-acetate, Alisol F, and Alismol against NAFLD and metabolic syndrome.* A. orientale* prevented hepatic triglyceride accumulation through suppressing de novo lipogenesis and increasing lipid export. In addition, it controlled oxidative stress markers, lipoapoptosis, liver injury panels, and inflammatory and fibrotic mediators, eventually influencing steatohepatitis and liver fibrosis. Moreover, it exhibited pharmacological activities against hyperlipidemia, obesity, and hyperglycemia as well as appetite. These biological actions of* A. orientale* might contribute to adiponectin activation or a role as a farnesoid X receptor agonist. In particular, Alisol A 24-acetate and Alisol B 23-acetate could be expected as main compounds. Taken together,* A. orientale* might be an effective candidate agent for the treatment of NAFLD and its comorbidities, although further assessment of its standardization, safety test, and clinical trials is consistently required.

## 1. Introduction

Nonalcoholic fatty liver disease (NAFLD), a new challenge of chronic liver disease in the 21^st^ century, includes simple steatosis, nonalcoholic steatohepatitis (NASH), fibrosis, and cirrhosis. A recent meta-analysis reported that global prevalence of NAFLD was assessed to be 25.24% [1], and its prevalence is likely to increase up to 33.5% in adults by 2030 [2]. As the prevalence of NAFLD constantly grows, economic burden is also predicted to consistently increase [3]. Most NAFLD patients have a high risk of cardiovascular disease-related mortality rather than liver-related death. Hence, NAFLD is not only a type of chronic liver diseases but also an independent risk factor of metabolic syndrome such as obesity, hypertension, type II diabetes mellitus (T2DM), and hyperlipidemia.

Unfortunately, there is still no gold standard medication to treat NAFLD. Pharmacological therapies for NAFLD currently depend on various options such as insulin sensitizing agents, antioxidants, incretin-based therapy, lipid lowering agents, and weight loss drugs other than lifestyle modification [4]. However, unfavorable side effects such as gastrointestinal upset, hemorrhagic stroke, myopathy, pruritus, osteoporosis, and transient increase in serum creatinine have hampered the authority approval as standard medication to treat NAFLD [5].

In the absence of optimal therapeutic strategies to approach NAFLD, herbal medicines containing abundant active substances could be an alternative and innovative therapeutic solution. Previous studies have demonstrated the brief but encouraging results on a total of 24 herbal plants against NASH [6, 7]. Among those plants,* Alisma orientale* (Sam.) Juz. (*A. orientale*) is worthy of notice.* A. orientale* is a synonym of* Alisma plantago-aquatica* subsp.* orientale* (Sam.) and belongs to the* Alisma* genus of the family of Alismataceae in the major group of Angiosperms. The tuber part of* A. orientale* contains various phytochemical constituents such as terpenoids, flavonoids, polysaccharides, phytosterols, and amino acids. Terpenoids including triterpenes, sesquiterpenes, and diterpenes are key compound classes of* A. orientale* contributing to its bioactive effects [8]. It has been mainly used for over 2000 years in Asian countries exhibiting diverse effects such as diuretic, hypolipidemic, hypoglycemic, antiallergic, and anti-inflammatory actions with no toxicity [8]. Numerous recent experimental studies suggested that* A. orientale* and its compounds exhibit therapeutic activities against NAFLD and its related comorbidities. Although* A. orientale* was introduced in the above two reviews, they were based on only three articles about the efficacy of* A. orientale* against NAFLD and its accompanied pathological diseases.

Therefore, this review summarizes preclinical evidence for* A. orientale* and its four constituents for the treatment of NAFLD and metabolic syndrome.

## 2. Pharmacological Effects of* A. orientale*

The pathological mechanism underlying the development and progression of NAFLD is complex and multifactorial. Thus, a ‘multiple hit' hypothesis provides a relatively accurate explanation about NAFLD pathogenesis. Such hits include insulin resistance, hormones secreted from the adipose tissue, oxidative stress, and inflammatory cytokines [9], which play important roles in the development of NAFLD and its progression. In addition, there exists an increasing evidence linking NAFLD with metabolic syndrome such as hyperlipidemia, obesity, and T2DM. Therefore, the pharmacological effects of* A. orientale* against NAFLD and metabolic syndrome could be discussed according to the following eight subthemes as antisteatotic, antioxidant, antilipoapoptotic, hepatoprotective, anti-inflammatory and antifibrotic, hypolipidemic, antiobesity, and hypoglycemic effects (Table 1).

### 2.1. Antisteatotic Activity

Hepatic steatosis, the hallmark of NAFLD, is characterized by TG accumulation in the hepatocyte cytoplasm, and regulating hepatic steatosis is an essential strategy to treat NAFLD and prevent its progression to NASH and hepatic inflammation. Currently, Aramchol™ (arachidyl-amino cholanoic acid) is an investigational anti-NASH drug suppressing TG levels and the activity of Stearoyl Coenzyme A Desaturase 1 (SCD1) involved in hepatic lipid accumulation [10].

Similarly,* A. orientale* inhibited overaccumulation of TG induced by free fatty acid (FFA) [11, 12], DL-ethionine [13], benzo(a)pyrene [14], high-fat diet [15–17], and tunicamycin [15] with accompanied lipid droplet decrease [11–13, 16]. Interestingly,* A. orientale* repressed the mRNA levels of Very Low-Density Lipoprotein (VLDL) receptor which accelerated hepatic TG overload in tunicamycin-treated HepG2 cells [15] and enhanced apoprotein B secretary protein supporting the excretion TG from hepatic cells in experimental models induced by DL-ethionine [13], tunicamycin, palmitate, and high-fat diet [15]. In addition,* A. orientale* could block hepatic lipid production by regulating hepatic lipogenic genes including fatty acid synthase (FAS), acetyl-coenzyme A carboxylase (ACC), and glycerol-3-phosphate acyltransferase (GPAT) [12, 15]. Taken together, these experimental results suggest that* A. orientale* could alleviate simple fatty hepatocytes via ER stress inhibition, hepatic lipogenesis suppression, and transfer of lipids out of liver.

### 2.2. Antioxidant Activity

Oxidative stress contributes to the pathological transition of simple hepatic steatosis to steatohepatitis and fibrosis. Particularly, oxidative stress markers such as thiobarbituric acid reactive substances (TBARS) or malondialdehyde (MDA) decrease beneficial antioxidant enzymes like superoxide dismutase (SOD) and cause overproduction of reactive oxygen species (ROS), secretion of proinflammatory cytokines, and influx of inflammatory monocytes into liver [18]. Consequently, ROS toxicity may activate Kupffer and hepatic stellate cells, leading to inflammation and fibrosis. Therefore, reducing oxidative stress and improving antioxidant defense system are essential to regulate the progression of NAFLD.

*A. orientale* administration elevated serum SOD activities [16] and reduced serum MDA levels in high-fat diet rats [16, 19]. In addition,* A. orientale* pretreatment significantly suppressed hepatic MDA formation induced by* tert*-butyl hydroperoxide in both HepG2 cells and rats [20]. Levels of ROS, TBARS, free radicals, and peroxides were also significantly reduced by* A. orientale* treatment in oxidative stress experimental models induced by palmitate [21] and* tert*-butyl hydroperoxide [20]. These results suggest that* A. orientale* has antioxidant effects to protect against liver damage initiated by oxidative stressors and it could be clinically applied as a therapeutic option to treat NAFLD patients.

### 2.3. Antilipoapoptotic Activity

Excessive nonesterified fatty acids (NEFAs) may induce lipotoxicity including the promotion of apoptosis. Thus, this lipid-induced apoptotic phenomenon is termed lipoapoptosis. Lipoapoptosis is a prominent feature of NASH and is associated with the severity and progression of NASH. In particular, c-Jun N-terminal kinase (JNK) phosphorylation is an important cause and mediator of lipoapoptosis in fibrosis as well as inflammation in the NASH liver [22]. Hence, blocking the JNK signaling pathway might be a beneficial strategy to treat NASH and prevent this progression.

In HepG2 cell models, free fatty acids treatment induced JNK-dependent lipoapoptosis by activating proapoptotic family including Bcl-2-associated X protein (Bax), caspase-3, and caspase-9 [11, 12, 21, 23]. Upon treatment of* A. orientale*, apoptotic mediators (Bax, Bcl-2, caspase-3, and caspase-9) and JNK activation were regulated resulting in a reduced number of apoptotic cells quantitatively [11, 12, 21, 23]. It is noteworthy that the mRNA and protein levels of p53 upregulated modulator of apoptosis (PUMA), a proapoptotic protein contributing to lipoapoptosis in hepatocytes, were suppressed by 100 *μ*g/ml of* A. orientale* treatment [11]. As JNK activation in NASH, PUMA is also overexpressed in the liver of NASH patients. In addition, PUMA is higher in liver tumor patients because it contributes to hepatocarcinogenesis [24] as well as lipoapoptosis [25]. JNK signaling is known to affect hepatic steatosis, insulin resistance, inflammation, fibrosis, and even cancer. Therefore,* A. orientale* could inhibit JNK-PUMA-mediated lipoapoptosis, which consequently might improve NASH and prevent its progression to fibrosis or HCC.

### 2.4. Hepatoprotective Activity

Simple hepatic steatosis is benign, but NASH is closely related to liver injury. Aspartate aminotransferase (AST) and alanine aminotransferase (ALT) are important biochemistry markers associated with liver injury. A large prospective UK cohort study suggested that NAFLD was the most prevalent cause of patients with abnormal AST and ALT levels [57]. In addition, fibrosis-4 index (age, platelet, ALT, and AST) and NAFLD fibrosis score (age, hyperglycemia, BMI, platelet, albumin, and AST/ALT ratio) including AST and ALT levels are calculated to predict the NASH progression and severity in clinical settings. Hence, normalizing AST and ALT levels is useful to treat NAFLD patients.

*A. orientale* exhibited hepatoprotective effects by lowering serum AST and ALT levels increased by high-fat diet in* in vivo* models [16, 17, 19, 26]. Moreover, the relatively high levels of AST and ALT in fatal liver injury rat models by benzo(a)pyrene [27], acetaminophen [28], and* tert*-butyl hydroperoxide [20] were significantly decreased after the administration of* A. orientale*. Consequently, it indicates that* A. orientale* could improve AST and ALT levels which are predictive of the presence of NAFLD or NASH and can be developed as a hepatoprotective agent like ursodeoxycholic acid to prevent its development and progression.

### 2.5. Anti-Inflammatory and Antifibrotic Activity

Inflammation and fibrosis are closely associated with the progress of hepatic simple steatosis to steatohepatitis and liver cirrhosis. In recent times, inflammatory and fibrotic mediators to treat NAFLD and prevent its progression are gaining attention for new therapeutic targets. Cenicriviroc, the dual antagonist of C-C chemokine receptor (CCR)2/CCR5 pathways in NASH-mediated inflammation and fibrosis, is currently being under phase III clinical trials for NASH patients with fibrosis (ClinicalTrials.gov Identifier: NCT03028740) [58].

As therapeutic targets for hepatic inflammation or fibrosis, tumor necrosis factor (TNF)-*α*, interleukin (IL)-6, and monocyte chemoattractant protein (MCP)-1 are representative cytokines known to correlate positively with severe NASH and advanced fibrosis [59, 60].* A. orientale* extract reduced the mRNA levels of TNF-*α*, IL-6, and MCP-1 increased in tunicamycin or palmitate-treated HepG2 cells and livers of high-fat diet or tunicamycin-injected mice [15]. Regarding proinflammatory cytokines, the protein expressions of cyclooxygenase (COX)-2 and inducible nitric oxide synthase (iNOS) were suppressed through the inhibition of phosphorylation of p65 subunit NF-*κ*B in NEFAs-treated HepG2 cells [12]. In addition, ER stress aggravates the inflammatory response through NF-*κ*B activation, but* A. orientale* prevented ER stress response by suppressing the mRNA expression of ER stress markers such as C/EBP homologous protein (CHOP), glucose-regulated protein 78 (GRP78), and X-box Binding Protein-1 (XBP-1) [15]. This activity of* A. orientale* against inflammation mediators could be explained by enhanced secretion of serum adiponectin by* A. orientale* administration in high-fat diet mice [26]. Adiponectin is one of important proteins involved in NAFLD pathogenesis and it exhibits anti-inflammatory actions by blocking NF-*κ*B and lowering the release of TNF-*α*, IL-6, COX-2, and iNOS. Additionally, adiponectin is known as an antifibrotic adipokine in the liver [61]. Consistently,* A. orientale* attenuated collagen deposition near the central veins and portal tracts in the liver in high-fat diet rats and tissue inhibitors of metalloproteinases (TIMP)-1 expression in hepatic stellate cells (HSC) [16, 29]. Collectively, although the specific mechanisms underlying these activities of* A. orientale* remain unclear, it likely influences the inflammatory and fibrogenic response of NAFLD and prevents the progression to NASH and fibrosis by regulating NF-*κ*B, adiponectin, and related markers.

### 2.6. Hypolipidemic Activity

Hypercholesterolemia results in hepatic cholesterol overload, in which cholesterol burden in liver brings about fatty liver. Moreover, cholesterol deposits activate resident macrophage, Kupffer cells, and subsequently lead to steatohepatitis. In particular, hydroxy-methylglutaryl CoA (HMG-CoA) reductase and acyl-CoA:cholesterol acyltransferase (ACAT) are two important enzymes affecting cholesterol synthesis and storage in liver. Hence, HMG-CoA inhibitor such as statin [62] or ACAT inhibitor like avasimibe [63] is possible in NAFLD patients for achieving cholesterol homeostasis.

*A. orientale* water extract showed comparatively high inhibition rates 18% and 13.08% against ACAT and HMG-CoA reductase activities in rat livers, respectively [30]. In addition,* A. orientale* lowered serum LDL levels in high-fat diet rats; increased LDL occurs due to overexpression of HMG-CoA reductase in NAFLD patients and it is a proatherosclerotic factor and a risk factor of NASH [19, 32]. Besides,* A. orientale* decreased serum TC and TG levels increased by high-fat diet intake as well as TC levels in the liver [14, 16, 17, 26]. A recent study reported that HDL and TG levels were more important causative factors of NASH than LDL or VLDL level [64]. The remarkable thing is that* A. orientale* increased the HDL serum levels and HDL/LDL ratio in high-fat diet rats [17, 26]. Elevated HDL is inversely proportional to cardiovascular disease which is the leading cause of the death in NAFLD patients. Collectively,* A. orientale* might regulate abnormal cholesterol-related markers which were indicative of NAFLD severity and progression and help prevent the development of cardiovascular diseases.

### 2.7. Antiobesity Activity

Obesity (BMI ≥ 30 kg/m^2^ in adults) is one of the risk factors resulting in the development and severity of NAFLD. Weight and BMI reduction could be one efficient intervention to treat obese NAFLD patients. However, around 8-19% of lean Asian (BMI < 25 kg/m^2^) are reported to have NAFLD [65] and this shows that BMI is an imperfect tool because it does not calculate muscle and fat mass separately. To approach the pharmacological treatment of obese and nonobese NAFLD patients, adipogenesis could be another therapeutic target as well as simple weight loss.

Administration of* A. orientale* extract markedly decreased not only body weight but also fat mass (abdominal subcutaneous fat, perirenal fat, and epididymal fat), which were increased by high-fat diet intake in* in vivo* models [16, 26, 31]. A histological finding showed that* A. orientale* water extract of 100 and 300 mg/kg reduced the size of adipocytes in fat tissue as compared with that in the normal diet group [26]. Consistently,* A. orientale* prevented adipocytes such as OP9 and 3T3-L1 cells from proliferating in number and differentiating into mature cells, leading to the inhibition of TG formation in differentiated 3T3-L1 adipocytes [33–35]. Concerning OP9 adipocyte differentiation, the expression of adipocyte-specific genes such as CCAAT enhancer binding protein (C/EBP)*β* (very early initiator of adipogenesis), C/EBP*α*, and peroxisome proliferator-activated receptor (PPAR)*γ* (essential transcriptional factors of adipogenesis) are suppressed by* A. orientale* treatment, suggesting that* A. orientale* might regulate adipogenic inducers [35]. In addition,* A. orientale* elevated the level of serum adiponectin [26], an important peptide hormone reduced in obese NAFLD patients [66], in mouse models, and ApoA-IV mRNA levels in intestinal cells. Importantly, elevated levels of ApoA-IV in the blood may reduce appetite for food by mediating hypothalamic melanocortin system [67]. These findings demonstrate that* A. orientale* may serve as an efficient drug to control food intake, reduce hyperplasia, hypertrophy, and differentiation of adipocytes and lose fat weight of obese and nonobese NAFLD patients. Currently, obese adults with NAFLD are more likely to increase the risk of drug-induced hepatotoxicity [68] and a randomized controlled trial identified that orlistat did not influence weight loss in NAFLD patients [69]. To overcome the limitation of oral medications for obese NAFLD patients,* A. orientale* extract and its chemical constituents could be applied to treat them because it has hepatoprotective effects as well as antiobesity effects.

### 2.8. Hypoglycemic Activity

Hepatic TG accumulation leads to insulin resistance and T2DM. Conversely, insulin resistance aggravates hepatic steatosis or inflammation. Most patients with NAFLD are at risk of the presence of insulin resistance. Although NAFLD patients with T2DM are exposed to increased risk of poor prognosis such as cardiovascular disease, liver cirrhosis, and HCC compared to those without T2DM [70], to date there is no specific drug approved to manage NAFLD and T2DM simultaneously. Currently, metformin is recommended as a safe drug in NASH and T2DM because it is excreted by renal clearance and not by liver metabolism. However, several meta-analyses showed that it is not efficient to improve NASH. Pioglitazone improved hepatic histological findings in necroinflammation and fibrosis but had some side effects such as weight gain or fluid retention [71]. This means that new therapeutic approaches to improve not only hyperglycemia but also NAFLD are required for NAFLD patients with T2DM.

*A. orientale* administration reduced the levels of serum glucose and HbA1c in in vivo diabetic models [16, 31, 37, 38, 41]. Suppressed brush border membrane vesicles (BBMV) (rabbit small intestine) intestinal glucose absorption and stimulated Hs68 (fibroblasts) and 3T3-L1 (adipocytes) glucose uptake by* A. orientale* treatment might be responsible for lowering glucose [36, 40]. In particular, the reduction of intestinal glucose absorption by* A. orientale* was supported by its inhibitory effect on *α*-glucosidase activity [40] which plays a key role in postprandial glycemic level through gastrointestinal absorption. Additionally,* A. orientale, *like TZD, increased insulin secretion and sensitivity through PPAR*γ* activation, contributing to its actions against hyperglycemia [16, 37, 39, 41]. Moreover,* A. orientale* enhanced the protein levels of insulin receptor substrate (IRS)-1 and protein kinase B (Akt) which are decreased in insulin resistant mice induced by high-fat diet and streptozotocin [41]. Subsequently,* A. orientale* suppressed the mRNA levels of hepatic PPAR*γ* coactivator (PGC)-1*α*, estrogen-related receptor (ERR)*γ*, and PGC-1*α*-dependent enzymes (G6Pase, phosphoenolpyruvate carboxykinase (PEPCK)) which are involved in gluconeogenesis in the liver tissue [26]. Furthermore,* A. orientale* elevated the serum adiponectin levels in high-fat diet mice [26] which is an important adipokine maintaining body and hepatic glucose homeostasis and preventing from progressing into inflammation and fibrosis. As mentioned above,* A. orientale* exhibited potential antidiabetic activity by regulating serum glucose, adiponectin, and insulin levels, hepatic/body insulin resistance, and excessive glucose production in the liver.

## 3. Pharmacological Effects of Active Constituents of* A. orientale*

**Figure 1:**
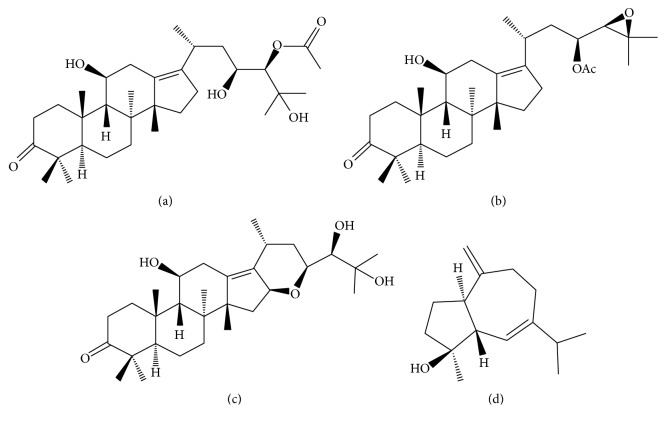
Chemical structures of constituents from* A. orientale*. (a) Alisol A 24-acetate (C_32_H_52_O_6_, molecular weight (MW) of 532.75 g/mol), (b) Alisol B 23-acetate (C_32_H_50_O_5_, MW of 514.8 g/mol), (c) Alisol F (C_30_H_48_O_5_, MW of 488.7 g/mol), and (d) Alismol (C_15_H_24_O, MW of 220.356 g/mol).

Chemical constituents of* A. orientale* are identified as about 120 compounds including guaiane-type sesquiterpenes, protostane-type triterpenes, guaiane-type and kaurane-type diterpenes [8], and small amounts of flavonoids, alkaloids, asparagine, phytosterols, fatty acids, and resins [72]. Protostane-type triterpenoids mainly include Alisols A–I and their derivatives while guaiane-type sesquiterpenoids include Alismol, Alismoxide, Orientalols A–F, and Orientalols sulphate [72]. In particular, experimental studies regarding the pharmacological activities of four compounds of Alisol A 24-acetate, Alisol B 23-acetate, Alisol F, and Alismol (Figure 1) among these various constituents of* A. orientale* have been increasingly reported. Hence, these four constituents were reviewed in this study in terms of their medicinal effects against NAFLD and its pathological process (Table 2).

### 3.1. Alisol A 24-Acetate

Triterpenes are currently regarded as one of the attractive phytochemical groups due to their therapeutic potential of anti-inflammatory, antiviral, antimicrobial, immunomodulatory, and antitumor actions [73]. Alisol A 24-acetate (Figure 1(a), C_32_H_52_O_6_, 532.75 g/mol) is one of the major active protostane-type triterpenes isolated from* A. orientale*. Alisol A 24-acetate decreased the lipid droplet, intracellular/hepatic TG and liver FFA contents accumulated by FFA or methionine and choline-deficient (MCD) in HepG2, WRL-68 human liver embryonic cell, and mouse models [42, 43, 45]. Simple hepatic steatosis is caused by excessive expression of TG synthetic genes (ACC and FAS) and reduced expression of carnitine palmitoyltransferase (CPT)1 and acyl-coA oxidase (ACOX)1 activating fatty acid oxidation. AMP-activated protein kinase (AMPK) activation suppressed TG accumulation in liver via inhibiting the activation of the sterol regulatory element-binding protein (SREBP)-1c transcriptional factor. The possible mechanism of Alisol A 24-acetate is likely through AMPK-SREBP-1c-FAS-ACC-CPT1-ACOX1 pathways against simple hepatic steatosis, and its intrinsic signaling may contribute to the antisteatotic effects of* A. orientale* [42]. In addition, adiponectin activation in the liver is known to be associated with the AMPK pathways [74]. Alisol A 24-acetate enhanced the adiponectin level in FFA-treated HepG2 cells and suppressed inflammatory cytokines (IL-6, IL-1*β*, MCP-1, and TNF-*α*) and fibrogenic factors (*α*-smooth muscle actin (SMA), transforming growth factor (TGF)-*β*, and TIMP) in NASH and fibrosis experimental models [42, 43]). Furthermore, Alisol A 24-acetate improved serum/liver lipid profile in hyperlipidemic mice induced by lipid emulsion diet and atherogenic diet [44, 45]. Collectively, Alisol A 24-acetate could be a key compound of* A. orientale* to contribute to its efficacy against NAFLD in the perspective of inhibiting the fundamental pathological process such as steatosis, inflammation, and fibrosis. Besides, a recent study reported that it effectively reversed the atherosclerotic markers, in particular, matrix metalloproteinase (MMP)-2/MMP-9 in smooth muscle cells [43]. Hence, Alisol A 24-acetate might be applied to manage a cardiovascular disease, a representative risk factor of NAFLD-related mortality.

### 3.2. Alisol B 23-Acetate

Alisol B 23-acetate (Figure 1(b), C_32_H_50_O_5_, 514.8 g/mol) is a major protostane triterpene which exhibits potent bioactivity. It is currently regarded as the official indicator for the quality control of medicinal herb* A. orientale* in the Pharmacopoeia of the People's Republic of China [72]. To date, emerging evidence demonstrates that Alisol B 23-acetate has a variety of therapeutic effects. In particular, its involvement with FXR deserves important results. Alisol B 23-acetate enhanced liver regeneration after partial resection [75] and maintained bile-acid homeostasis [76], both via FXR activation. Its role as FXR agonist is expanded to its protective activities against NASH in mice. Indeed, it played a critical action in TG and fatty acid synthesis and metabolism, thus preventing inflammation and fibrogenesis in MCD-diet mice via FXR stimulation [46]. Its antioxidant [47, 77] and hepatoprotective [46] effects are also likely associated with downstream target genes of FXR. Hence, Alisol B 23-acetate is anticipated to show pharmacological effects similar to FXR agonists such as obeticholic acid and ursodeoxycholic acid in NAFLD treatment. In addition, it exhibited strong hypolipidemic effects by inhibiting the activity of HMG-CoA reductase and activating lipoprotein lipase (LPL) activity. In particular, its binding interaction with HMG-CoA reductase is even more potent than Alisol A 24-acetate [44]. Taken together, Alisol B 23-acetate might substantially contribute to the biological actions of* A. orientale* against NAFLD based on its effect of downregulating hepatic lipid genesis, increasing lipid output, regulating inflammation and fibrosis, and exerting hepatoprotective effects via FXR activation.

### 3.3. Alisol F

Alisol F (Figure 1(c), C_30_H_48_O_5_, 488.7 g/mol) is one of protostane-type triterpenes isolated from* A. orientale* like above two compounds. A previous study reported that it exhibited antiviral activity against hepatitis B virus in HepG2.2.15 cells with the inhibitory concentration (IC)_50_ of 0.6 *μ*M and 8.5 *μ*M on HBsAg and HBeAg secretion, respectively [78]. Apart from its antiviral effect, Alisol F has been mainly investigated to check its pharmacological activities against inflammation. Alisol F suppressed a variety of powerful inflammatory mediators such as iNOS, NO, COX-2, TNF-*α*, IL-6, and IL-1*β* elevated by lipopolysaccharide (LPS) in macrophages and LPS or D-*gal *injection in mice [48–50]. The molecular mechanism of Alisol F against LPS-induced inflammation is reported to involve activation of NF-*κ*B and phosphorylation of its upstream molecules mitogen-activated protein kinase (MAPK)s (extracellular-signal-regulated kinase (ERK), p38, JNK) [48]. Additionally, Alisol F alleviated acute hepatic failure induced by LPS and D-*gal *injection by lowering AST and ALT levels in mice [48]. Besides, Alisol F could regulate hyperglycemia via *α*-glucosidase activity inhibition without adipose cell differentiation and lipogenesis unlike thiazolidinedione (TZD)s [40]. However, evidence on the effects of Alisol F against NAFLD is lacking and further study is needed to elucidate its pharmacological activities.

### 3.4. Alismol

Alismol (Figure 1(d), C_15_H_24_O, 220.356 g/mol) is one of guaiane-type sesquiterpenes isolated from* A. orientale* that possesses anti-inflammatory activity by inhibiting NO production and iNOS synthesis in RAW 264.7 cells induced by LPS [50, 51]. In addition, Alismol showed significant blocking effects against the GRP78 expression, a ER stress marker, in tunicamycin-treated HepG2 cells [15]. Tunicamycin is a sort of ER stress inducer by causing the unfolded protein response (UPR) in cells. UPR and ER stress are often observed in hepatic cells of NAFLD or obesity patients and may play a pivotal role in the progression to NASH or cirrhosis. Since GRP78 leads to the UPR survival, the pharmacological effects suppressing the GRP78 expression of Alismol suggest that it might be one of representative compounds of* A. orientale* contributing to anti-ER stress and hepatic steatosis [15]. Interestingly, Alisol B 23-acetate, a triterpenoid of* A. orientale* did not show protective effects against ER stress marker proteins. Therefore, further study of therapeutic and preventive effects of* A. orientale* against NAFLD and its progression needs to be implemented using Alismol as well as Alisol B 23-acetate. Furthermore, Alismol was found to exhibit antihypertensive effects via the inhibition of sympathetic neuron and Ca^2+^ influx [50, 52–55], which might be interconnected with the diuretic activities of* A. orientale*. Since hypertension is a risk factor of cardiovascular disease which increases the mortality of NAFLD patients, Alismol is worthy of attention for the treatment of NAFLD.

## 4. Discussion

**Figure 2:**
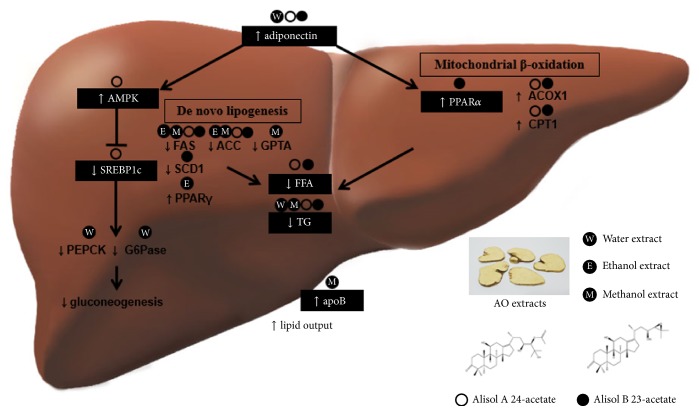
Molecular mechanism related to pharmacological effects of* A. orientale* regulating lipid and glucose metabolism in liver.* A. orientale* stimulated adiponectin and subsequently suppressed hepatic de novo lipogenesis and accelerated fatty acid oxidation via AMPK and PPAR*α* activation, resulting in decreased hepatic TG contents and lipid output acceleration from liver. In addition,* A. orientale* regulated hepatic gluconeogenesis by lowering PEPCK and G6Pase mRNA via AMPK-SREBP1c signaling.

This is the first review of* A. orientale* actions and its molecular mechanisms against NAFLD and metabolic syndrome. First,* A. orientale* including Alisol A 24-acetate and Alisol A 23-acetate hindered hepatic de novo lipogenesis and accelerated *β*-oxidation via AMPK and PPAR*α* activation by adiponectin, leading to the inhibition of hepatic TG accumulation and increase of lipid output from liver. In addition,* A. orientale* suppressed hepatic gluconeogenesis by regulating hepatic expression of glucogenic genes like PEPCK and G6Pase via AMPK-SREBP1c signaling (Figure 2). This antidiabetic effect of* A. orientale* could be influenced by its actions elevating adiponectin. Adiponectin not only is a key cytokine for NAFLD but also is involved in obesity, T2DM, inflammation, apoptosis, fibrosis, and even cancer. Adiponectin has been described as an ideal target against NAFLD. TZDs, approved T2DM drugs, are currently regarded as replaceable agents for targeting adiponectin in NAFLD patients, but there exist some limitations of weight gain or insignificant lobular inflammation, ballooning, and fibrosis improvement [79]. These results stimulate the further investigation of* A. orientale*.

Second,* A. orientale* is expected to exhibit pharmacological effects against NAFLD and metabolic syndrome similar to an FXR agonist. Alisol B 23-acetate intervened the downstream regulators of FXR such as SREBP1c, PPAR*α*, and genes involved in triglyceride metabolism (ApoC-II, ApoC-III, and angiopoietin like ANGPTL3), contributing to the improvement of hyperlipidemia as well as hepatic steatosis. In addition,* A. orientale* reversed cholestasis, AST, and ALT levels by activating FXR. FXR is a transcriptional factor mainly expressed in the liver, intestine, and kidney. Since patients with NAFLD have decreased hepatic expression of protein and mRNA of FXR and it is associated with hepatic steatosis, inflammation, fibrosis, injury, and even cancer [80], FXR appears to gain increasing interest as a promising target to treat NAFLD. Although obeticholic acid is a representative FXR agonist, hyperlipidemia and hyperglycemia are well-known unfavorable effects [81]. Hence, it needs further study about AO to elucidate its role as a FXR agonist and check its possible side effects for the treatment of NAFLD and metabolic syndrome.

Based on most studies, Alisol A 24-acetate and Alisol B 23-acetate could be key bioactive components of* A. orientale* against NAFLD and metabolic syndrome. However, these compounds were not obtained from the water extract of* A. orientale* which is prevalent in making herbal decoctions, and it is difficult to use methanol extract of* A. orientale* in practical use because of safety concerns from methanol extraction process. Meanwhile, water extract of* A. orientale* exhibited antisteatotic, antioxidant, antilipoapoptotic, hepatoprotective, anti-inflammatory and antifibrotic, hypolipidemic, antiobesity, and hypoglycemic effects. Therefore, identification for efficacy component from water extract of* A. orientale* needs to be established using systematic novel analysis to show the correlation between component contents and bioactivity for the quality control of* A. orientale*.

## 5. Conclusion

Despite ongoing studies, there still exist difficulties in identifying specific pathophysiological mechanism underlying NAFLD and metabolic syndrome. This is the first review to demonstrate in detail that* A. orientale* and its chemical substances can contribute to the treatment of NAFLD and metabolic syndrome based on their pharmacological activities such as antisteatotic, antioxidant, antilipoapoptotic, hepatoprotective, anti-inflammatory, antifibrotic, hypolipidemic, antiobesity, and hypoglycemic effects. In particular,* A. orientale* regulated effectively lipid and glucose metabolism in the liver and controlled liver injury like oxidative stress, inflammation, and fibrosis. Moreover,* A. orientale* was involved in hyperglycemia, obesity, or hyperlipidemia, representative comorbidities of NAFLD. The underlying mechanism of* A. orientale* is partly revealed to be linked to adiponectin, AMPK, SREBP1c, or FXR. In particular, Alisol A 24-acetate and Alisol B 23-acetate are considered main effective compounds of* A. orientale*. In addition, since* A. orientale* strengthened the ApoA-IV promoter activity and elevated its mRNA level in intestinal cells, more supplementary data and efforts to identify effective compounds on the control of food intake are required to support the potential of* A. orientale*. With a comprehensive approach, this review is anticipated to support pharmacological activities of* A. orientale* and its compounds and serve as a stimulus to develop novel therapeutic and preventive drugs against NAFLD and metabolic syndrome. Further studies are needed to verify valid ingredients, efficient dosage, and extraction procedures of* A. orientale* to maximize its therapeutic potential. Moreover, a variety of preclinical results in this review are required to be driven to more reasonable conclusion through a well-designed and appropriate clinical trials for potential clinical application of* A. orientale*.

## Figures and Tables

**Table 1:** Pharmacological properties of *A. orientale*.

Extraction solvent	Country	Type	Model	Efficient doses	Results	References
*Antisteatotic activity*						

Water	China	*In vitro*	DL-ethionine-treated rat hepatocytes	1, 5, 10, 20, and 50 *μ*g/ml	Apolipoprotein B ↑ liver TG ↓ lipid droplet ↓	[13]
		*In vivo*	High-fat diet rats	2.26 g/kg	Liver TG↓	[17]

Ethanol	South Korea	*In vitro*	FFA-treated HepG2	100 *μ*g/ml	Lipid droplet ↓	[11]
		*In vitro*	NEFAs-treated HepG2	300 *μ*g/ml	Lipid droplet ↓ FAS mRNA & protein ↓ ACC mRNA & protein ↓	[12]

Methanol	China	*In vivo*	High-fat diet rats	300, 600 mg/kg	Liver weight↓ Liver weight/body weight ratio↓ Liver TG ↓ lipid droplet ↓	[16]
	South Korea	*In vivo*	Benzo(a)pyrene-injected rats	0.15 g/kg	Liver TG↓	[14]
	South Korea	*In vitro*	Tunicamycin-treated HepG2	10, 50, and 100 *μ*g/ml	TG ↓ VLDL receptor ↓ Apolipoprotein B ↑	[15]
		*In vitro*	Palmitate -treated HepG2	10, 50, and 100 *μ*g/ml	Hepatic lipogenic genes (FAS, ACC, and GPAT) ↓ TG ↓ VLDL receptor ↓Apolipoprotein B ↑	
		*In vivo*	Tunicamycin-injected mice	50, 100 mg/kg	Liver TG ↓VLDL receptor ↓Apolipoprotein B ↑	
		*In vivo*	High-fat diet mice	100, 300 mg/kg	Hepatic lipogenic genes ↓liver TG ↓VLDL receptor↓ Apolipoprotein B ↑	

*Antioxidant activity*						

Water	South Korea	*In vitro*	Palmitate -treated HepG2	100 *μ*g/ml	ROS ↓ TBARS ↓	[21]

Ethanol	South Korea	*In vitro*	tert-Butyl hydroperoxide-induced HepG2	0.05, 0.1 mg/ml	Free radicals ↓ Superoxide anions ↓ MDA ↓	[20]
		*In vivo*	tert-Butyl hydroperoxide-induced rats	1 g/kg	Liver MDA ↓	

Methanol	China	*In vivo*	High-fat diet rats	300, 600 mg/kg	Serum MDA ↓ Serum SOD ↑	[16]
	South Korea	*In vivo*	High-fat diet rats	100, 200, and 300 mg/kg	Serum MDA ↓	[19]

*Antilipoapoptotic activity*						

Water	South Korea	*In vitro*	Palmitate -treated HepG2	100 *μ*g/ml	Apoptotic cells ↓ sub-G1 cells ↓ BAX ↓ Bcl-2 ↑ pJNK ↓	[21]
	South Korea	*In vitro*	Palmitate -treated HepG2	10, 100 *μ*g/ml	Sub-G1 cells ↓	[23]

Ethanol	South Korea	*In vitro*	NEFAs-treated HepG2	300 *μ*g/ml	MAPK8 mRNA ↓ p-JNK ↓ BAX ↓ Bcl-2 ↑ Cleaved caspase-9 ↓ Cleaved caspase-3↓	[12]
	South Korea	*In vitro*	FFA-treated HepG2	100 *μ*g/ml	p-JNK ↓ PUMA mRNA & protein ↓ BAX ↓ Bcl-2 ↑ Cleaved caspase-3 ↓ Cleaved caspase-9 ↓	[11]

*Hepatoprotective activity*						

Water	South Korea	*In vivo*	High-fat diet mice	100, 300 mg/kg	Serum AST ↓ Serum ALT ↓	[26]
	China	*In vivo*	High-fat diet mice	2.26 g/kg	Serum AST ↓ Serum ALT ↓	[17]
	South Korea	*In vivo*	Benzo(a)pyrene-injected rats	9 g/kg	Serum AST ↓ Serum ALT ↓ Liver AST ↓ Liver ALT ↓	[27]

Ethanol	South Korea	*In vivo*	tert-Butyl hydroperoxide-induced rats	1 g/kg	Serum AST ↓ Serum ALT ↓	[20]

Methanol	China	*In vivo*	High-fat diet rats	150, 300, and 600 mg/kg	Serum AST ↓ Serum ALT ↓	[16]
	South Korea	*In vivo*	High-fat diet rats	100, 200, and 300 mg/kg	Serum AST ↓ Serum ALT ↓	[19]
	South Korea	*In vivo*	Acetaminophen-injected rats	250, 500 mg/kg	Serum AST ↓ Serum ALT ↓	[28]

*Anti-inflammatory and antifibrotic activity*						

Water	South Korea	*In vivo*	High-fat diet mice	100 mg/kg	Serum adiponectin↑	[26]

Ethanol	South Korea	*In vitro*	NEFAs-treated HepG2	300 *μ*g/ml	NF-*κ*B p65(p65) ↓ p-p65 ↓ COX-2 ↓ iNOS ↓	[12]
	South Korea	*In vitro*	Human hepatic stellate cells	0.02, 0.1 mg/ml	TIMP-1↓	[29]

Methanol	South Korea	*In vitro*	Tunicamycin-treated HepG2	10, 50, and 100 *μ*g/ml	GRP78 mRNA↓ CHOP mRNA↓ XBP-1 mRNA↓ IL-6 mRNA ↓ TNF-*α* mRNA↓ MCP-1 mRNA ↑	[15]
		*In vitro*	Palmitate -treated HepG2	10, 50, and 100 *μ*g/ml		
		*In vivo*	Tunicamycin-injected mice	50, 100 mg/kg	Liver GRP78 mRNA↓ liver CHOP mRNA↓ liver XBP-1 mRNA↓ liver IL-6 mRNA ↓ Liver TNF-*α* mRNA↓ liver MCP-1 mRNA ↑	
		*In vivo*	High-fat diet mice	100, 300 mg/kg		
	China	*In vivo*	High-fat diet rats	150, 300, and 600 mg/kg	Liver collagen deposition↓	[16]

*Hypolipidemic activity*						

Water	South Korea	*In vitro*	Microsome from rat liver	10 *μ*l	Liver ACAT↓ Liver HMA-CoA reductase↓	[30]
	South Korea	*In vivo*	High-fat diet mice	100 mg/kg	Serum TG ↓ Serum TC ↓ Serum LDL ↓ Serum HDL ↑ Serum HDL/LDL ↑	[26]
	China	*In vivo*	High-fat diet rats	2.26 g/kg	Serum TC ↓ Serum TG ↓ Liver TC ↓ Serum HDL ↑ Liver HMG-CoA reductase ↓	[17]

Methanol	China	*In vivo*	High-fat diet rats	300, 600 mg/kg	Serum TC ↓ Serum TG ↓ Liver TC ↓	[16]
	South Korea	*In vivo*	Benzo(a)pyrene-injected rats	0.15 g/kg	Serum TG↓ Serum TC↓ liver TC↓	[14]
	South Korea	*In vivo*	High-fat diet rats	100, 200, and 300 mg/kg	Serum LDL ↓	[19]

*Antiobesity activity*						

Water	China	*In vivo*	Goto-Kakizaki rats	3 mg/g	Body weight↓	[31]
	South Korea	*In vivo*	High-fat diet mice	100 mg/kg	Body weight↓ Total fat weight/ Body weight↓ Adipocyte size↓ Serum Adiponectin↑	[32]
	South Korea	*In vitro*	3T3-L1 cells	10 mg/ml	Proliferation↓ Differentiation↓	[33]
	China	*In vitro*	Caco-2/TC7 transfected with human ApoA-IV promoter	1 mg/ml	ApoA-IV promoter activity↑ ApoA-IV mRNA↑	[34]
			3T3-L1 cells	1, 10 mg/ml	TG↓	

Ethanol	South Korea	*In vitro*	OP9 cells	20, 40 *μ*g/ml	PPAR*γ* protein↓ PPAR*γ* mRNA↓ C/EBP*α* mRNA↓ C/EBP*β* protein↓	[35]

Methanol	China	*In vivo*	High-fat diet rats	300, 600 mg/kg	Epididymal fat weight ↓ Epididymal fat weight/body weight↓	[16]

*Hypoglycemic activity*						

Water	China	*In vitro*	BBMV	1 mg/ml	Intestinal glucose absorption ↓	[36]
			Hs68 cells	0.01, 0.1, and 1 mg/ml	Fibroblast glucose uptake ↑	
			3T3-L1 cells	0.01, 0.1, and 1 mg/ml	Adipocyte glucose uptake ↑	
	China	*In vivo*	Streptozotocin-induced mice	1.5, 3 g/kg	Serum glucose ↓ Serum insulin ↑	[37]
	South Korea	*In vivo*	Streptozotocin-induced rats	61.25 mg/kg	Serum glucose ↓	[38]
	China	*In vivo*	Goto-Kakizaki rats	3 mg/g	Fasting serum glucose ↓ glucose tolerance ↑	[31]
	South Korea	*In vivo*	High-fat diet mice	100 mg/kg	Serum adiponectin↑ Liver PGC-1*α*, ERR*γ*, G6Pase, PEPCK mRNA↓	[26]

Ethanol	South Korea	*In vitro*	3T3-L1 cells	50 *μ*g/ml	PPAR*γ* agonist activity ↑	[39]

Methanol	China	*In vivo*	High-fat diet rats	300, 600 mg/kg	Fasting serum glucose ↓ insulin sensitivity index ↑ Insulin resistance index ↓	[16]

Alcohol	China	*In vitro*	3T3-L1 cells	25, 50, and 100 *μ*g/ml	Adipocyte glucose uptake ↑	[40]
			*α*-Glucosidase assay	25 *μ*g/ml	*α*-Glucosidase activity ↓	

Ethyl acetate	China	*In vivo*	High-fat diet and streptozotocin-induced mice	100 mg/kg	Fasting serum glucose ↓ serum insulin ↑ serum HbA1c ↓ IRS-1 protein ↑ Akt protein ↑	[41]

**Table 2:** Pharmacological activities of Alisol A 24-acetate, Alisol B 23-acetate, Alisol F, and Alismol.

Pharmacological effects	Country	Type	Model	Doses	Results	References
*Alisol A 24-acetate*						

Antisteatotic	China	*In vitro*	FFA-treated HepG2	10 *μ*M	Lipid droplet↓ FAS, ACC, AMPK, SREBP-1c mRNA & protein ↓ CPT1, ACOX1 mRNA & protein ↑	[42]
	China	*In vitro*	MCD-treated WRL-68	1, 2, 4, 8, and 16 *μ*M	TG↓	[43]
		*In vivo*	MCD diet mice	60 mg/kg	Lipid droplet↓ Liver TG↓ Liver FFA↓	
Antioxidant	China	*In vivo*	MCD diet mice	60 mg/kg	Liver ROS, MDA, MPO↓	
		*In vitro*	LX-2	4, 8 *μ*M	ROS↓	
Hepatoprotective	China	*In vivo*	MCD diet mice	30, 60 mg/kg	Serum AST↓ Serum ALT↓	
Anti-inflammatory	China	*In vivo*	MCD diet mice	60 mg/kg	Liver inflammatory foci↓ IL-6↓ IL-1*β*↓ MCP-1↓	
		*In vitro*	LX-2	4, 8 *μ*M	IL-6, IL-1*β*, MCP-1 mRNA↓	
		*In vitro*	FFA-treated HepG2	10 *μ*M	TNF-*α* ↓ IL-6 ↓ Adiponectin ↑	[42]
Antifibrotic	China	*In vivo*	MCD diet mice	60 mg/kg	Liver extracellular matrix↓ *α*-SMA↓ TGF-*β*↓ TIMP↓	[43]
		*In vitro*	LX-2	8, 16 *μ*M	*α*-SMA, TGF-*β* mRNA&protein↓ TIMP mRNA↓	
Hypolipidemic	China	*In vivo*	Lipid emulsion diet mice	0.64, 1.28, and 2.56 mg/kg	Serum TC, TG, LDL, HDL↓ liver HMG-CoA reductase ↓	[44]
	Japan	*In vivo*	Atherogenic diet rats	97.5 mg/kg	Serum TC↓ Liver fat↓ Liver TC↓	[45]
Antiobesity	China	*In vitro*	FFA-treated HepG2	10 *μ*M	Adiponectin ↑	[42]
Hypoglycemic	China	*In vitro*	FFA-treated HepG2	10 *μ*M	Adiponectin ↑	

*Alisol B 23-acetate*						

Antisteatotic	China	*In vivo*	MCD diet mice	15, 30, and 60 mg/kg	Lipid droplet↓ liver TG, FFA↓ FAS, ACC, SCD1 protein↓ CPT1, ACOX1 mRNA↑ PPAR*α* mRNA↑	[46]
Antioxidant	South Korea	*In vivo*	Bromobenzene -injected rats	10, 20 mg/kg	Liver MDA, glutathione↓liver Glutathione↓	[47]
Hepatoprotective	China	*In vivo*	MCD diet mice	15, 30, and 60 mg/kg	Serum AST↓ Serum ALT↓	[46]
Anti-inflammatory	China	*In vivo*	MCD diet mice	30, 60 mg/kg	Serum MCP-1↓ mouse keratinocyte-derived chemokine↓	
				60 mg/kg	liver MCP-1, VCAM-1 mRNA↓	
Antifibrotic	China	*In vivo*	MCD diet mice	60 mg/kg	a1(I), a2(I) collagen mRNA↓ *α*-SMA, TGF-*β*, MMP-2, TIMP-1 mRNA↓	
Hypolipidemic	China	*In vivo*	Lipid emulsion diet mice	0.64, 1.28, 2.56 mg/kg	Serum TC, TG, LDL, HDL↓ liver HMG-CoA reductase ↓	[44]
	China	*In vivo*	MCD diet mice	30, 60 mg/kg	Serum TG, FFA, TC↓ liver TC↓ LPL mRNA↑ ApoC-II mRNA↑ ApoC-III mRNA↓ ANGPTL3 mRNA↓	[46]

*Alisol F*						

Hepatoprotective	China	*In vivo*	LPS/D-gal-induced mice	20 mg/kg	Serum AST↓ Serum ALT↓	[48]
Anti-inflammatory	China	*In vitro*	LPS-treated RAW264.7	3.7, 11, and 33 *μ*M	iNOS, COX-2 mRNA & protein↓ TNF-*α*, IL-6, IL-1*β* mRNA & protein↓ NF-*κ*B↓ MAPKs(ERK, p38, JNK)↓ STAT3↓	
		*In vivo*	LPS/D-gal-induced mice	20 mg/kg	Serum TNF-*α*, IL-6, IL-1*β* ↓ liver MAPKs(ERK, JNK)↓	
	China	*In vitro*	LPS-treated RAW264.7	3.7, 11, and 33 *μ*M	NO↓	[49]
	Japan	*In vitro*	LPS-treated macrophages	50, 100 *μ*M	NO↓ iNOS↓	[50]
Hypoglycemic	China	*In vitro*	3T3-L1 cells	10 *μ*M	Cell differentiation↓	[40]
			*α*-Glucosidase assay	0.125, 0.25, 0.5, 1, and 2.5 mM	*α*-Glucosidase activity↓	

*Alismol*						

Anti-inflammatory	South Korea	*In vitro*	Tunicamycin-treated HepG2	100 *μ*M	GRP78 mRNA↓	[15]
	China	*In vitro*	LPS-treated RAW264.7	0.39, 1.56, 6.25, 25, and 100 *μ*M	NO↓	[51]
	Japan	*In vitro*	LPS-treated macrophages	50, 100 *μ*M	NO↓ iNOS↓	[50]
Antihypertensive	Japan	*In vivo*	Hypertensive rats	100 mg/kg	Blood pressure↓	[52]
	Japan	*In vivo*	Heparin-treated rats	10 mM	Cardiac output↓ Heart rate↓ Left ventricular pressure↓ Coronary flow↑	[53]
	Japan	*In vitro*	Ca2+-treated rabbit thoracic aorta tissue	10, 300 mM	Contractile response↓	[54]
	Japan	*In vitro*	Angiotensin I-treated rabbit thoracic aorta tissue	10 mM	Contractile response↓	[55]
	Japan	*In vitro*	Noradrenaline	10 mM	Contractile response↓	[56]
